# 2893 A. Efficacy and Safety of Aztreonam-Avibactam for the Treatment of Serious Infections Due to Gram-Negative Bacteria, Including Metallo-β-Lactamase-Producing Pathogens: Phase 3 REVISIT Study

**DOI:** 10.1093/ofid/ofad500.2476

**Published:** 2023-11-27

**Authors:** Yehuda Carmeli, Jose-Miguel Cisneros, Mical Paul, Georgios L Daikos, Minggui Wang, Julian Torre Cisneros, George Singer, Ivan Titov, Illia Gumenchuk, Yongjie Zhao, Rosa-María Jiménez Rodríguez, Lu Liang, Gang Chen, Oleksandr Pyptiuk, Firdevs Aksoy, Halley Rogers, Michele Wible, Francis Arhin, Alison Luckey, Joanne Leaney, Rienk Pypstra, Joseph Chow

**Affiliations:** Israel Ministry of Health, Tel Aviv, Tel Aviv, Israel; Virgen del Rocío University Hospital-IBiS, Seville, Andalucia, Spain; Rambam Health Care Campus, Haifa, Hefa, Israel; laiko General Hospital, Athens, Attiki, Greece; Institute of Antibiotics, Huashan Hospital, Fudan University, Shanghai, Shanghai, China (People's Republic); Hospital Universitario Reina Sofia, Córdoba, Andalucia, Spain; Harbor-UCLA Medical Center, Torrance, California; Ivano-Frankivsk National Medical University, Ivano-Frankivsk, Ivano-Frankivs'ka Oblast', Ukraine; Communal Non-profit Enterprise Vinnytsia Regional Clinical Hospital Named after M.I.Pyrogov Vinnytsia Regional Council, Vinnytsia, Vinnyts'ka Oblast', Ukraine; Tianjin Union Medical Center, Tianjin, Hebei, China; Virgen del Rocío University Hospital-IBiS, Seville, Andalucia, Spain; Baotou Central Hospital, Baotou, Nei Mongol, China; The First Hospital of Kunming, Kunming, Yunnan, China; Ivano-Frankivsk National Medical University, Ivano-Frankivsk, Ivano-Frankivs'ka Oblast', Ukraine; Karadeniz Technical Univ., Trabzon, Trabzon, Turkey; Pfizer Clinical Development & Operations, New York, New York; Pfizer WW Research & Development, Collegeville, Pennsylvania; Pfizer, Inc., Kirkland, Manitoba, Canada; Global Antibiotic R&D Partnership, Geneva, Geneve, Switzerland; Pfizer WW Research & Development, Collegeville, Pennsylvania; Pfizer Inc, New York, New York; Pfizer Global Product Development, Collegeville, Pennsylvania

## Abstract

**Background:**

Multidrug resistant (MDR) Gram-negative bacteria, including metallo-β-lactamase (MBL) producers, pose significant treatment challenges. This study investigated efficacy and safety of aztreonam-avibactam (ATM-AVI) in the treatment of complicated intra-abdominal infection (cIAI) or hospital-acquired)/ventilator-associated pneumonia (HAP/VAP) due to Gram-negative bacteria, including MBL-producing MDR pathogens, with limited or no treatment options.

**Methods:**

REVISIT was a phase 3, prospective, randomized, multicenter, open-label, central assessor-blinded study in hospitalized adults. Patients were randomized 2:1 to ATM-AVI (± metronidazole [MTZ]; cIAI patients only) or meropenem (MER) ± colistin (COL) for 5–14 (cIAI) or 7–14 (HAP/VAP) days. Clinical cure at the test-of-cure (TOC) visit in the intent-to-treat (ITT) and clinically evaluable (CE) analysis sets were the primary efficacy endpoints. Key secondary endpoints included microbiological responses at TOC, 28-day mortality, and safety. No formal hypothesis testing was planned.

**Results:**

In total, 422 patients were randomized (ATM-AVI ± MTZ, n=282; MER ± COL, n=140). Adjudicated clinical cure rates at TOC are shown in Table 1. Favorable microbiological response rates at TOC (micro-ITT analysis set) were 75.7% for ATM-AVI ± MTZ and 73.9% for MER ± COL; 28-day all-cause mortality rates for ATM-AVI ± MTZ and MER ± COL were 1.9% (4/208) vs 2.9% (3/104), and 10.8% (8/74) vs 19.4% (7/36) in cIAI and HAP/VAP, respectively. Adverse events (AEs) are summarized in Table 2. There were no treatment-related serious AEs in the ATM-AVI group.

**Figure:**
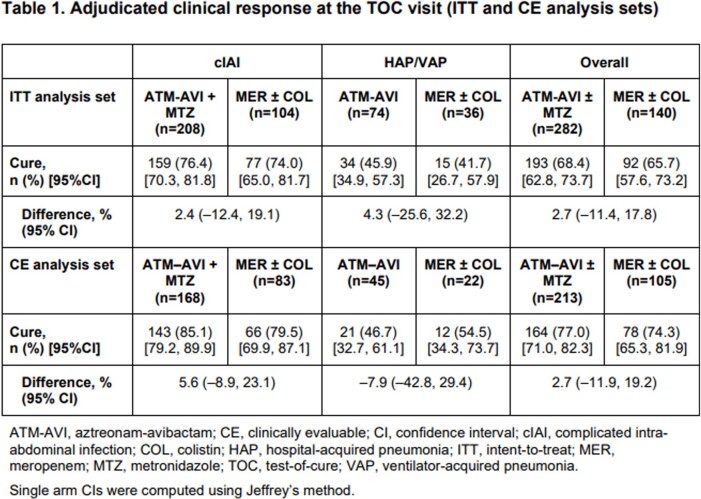

**Figure:**
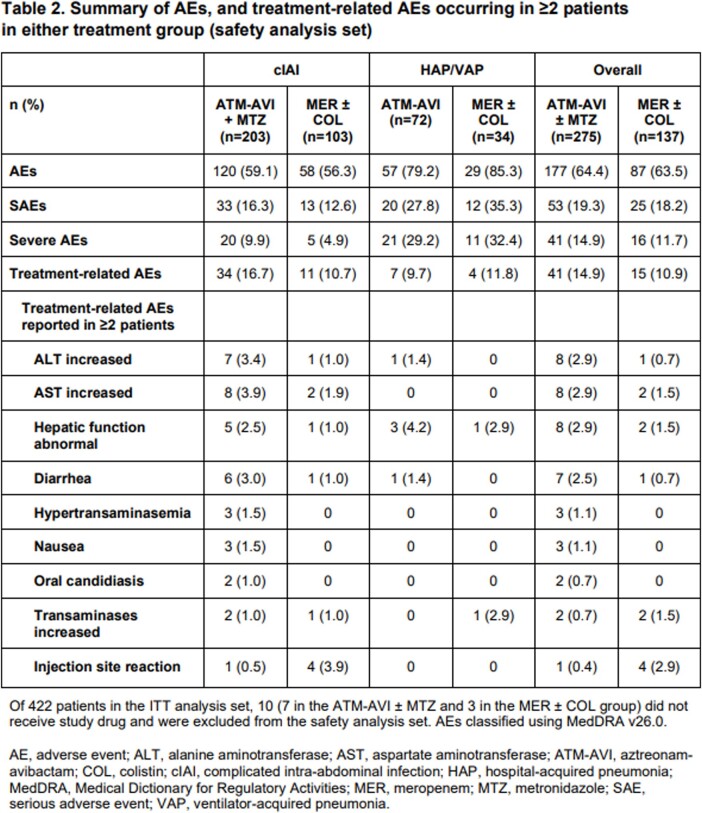

**Conclusion:**

ATM-AVI (± MTZ) was effective in treating patients with cIAI and HAP/VAP, displaying similar efficacy to MER ± COL. ATM-AVI was generally well tolerated. These data support potential use of ATM-AVI for the treatment of serious infections caused by susceptible Gram-negative bacteria. Further analyses will focus on MBL-producing pathogens.

***Trial registration.*** NCT03329092. Study sponsored by Pfizer. ATM-AVI is jointly developed with AbbVie, also supported by the United States Biomedical Advanced Research and Development Authority (BARDA) and the European Innovative Medicines Initiative (IMI), under the COMBACTE-CARE consortium.

**Disclosures:**

**Yehuda Carmeli, MD**, Merck: Advisor/Consultant|Merck: Grant/Research Support|Merck: Honoraria|Pfizer: Advisor/Consultant|Pfizer: Grant/Research Support|Pfizer: Honoraria|Qpex: Advisor/Consultant|Qpex: Grant/Research Support|Qpex: Honoraria|Roche: Advisor/Consultant|Roche: Grant/Research Support|Roche: Honoraria **Georgios L. Daikos, PhD**, MSD: Honoraria|Pfizer: Advisor/Consultant|Pfizer: Honoraria|Viatris: Honoraria **Rosa-María Jiménez Rodríguez, MD, PhD**, Abex: Honoraria|B. Braun: Honoraria|Johnson & Johnson: Honoraria **Halley Rogers, MPH**, Pfizer: Ownership Interest **Michele Wible, MS**, Pfizer: Ownership Interest **Francis Arhin, PhD**, Pfizer: Ownership Interest **Joanne Leaney, PhD**, Pfizer: Ownership Interest **Rienk Pypstra, MD, MBA**, Pfizer: Stocks/Bonds **Joseph Chow, MD**, Pfizer: Ownership Interest

